# Increased response to TPF chemotherapy promotes immune escape in hypopharyngeal squamous cell carcinoma

**DOI:** 10.3389/fphar.2022.1097197

**Published:** 2023-01-13

**Authors:** Ruichen Li, Li Yan, Shu Tian, Yang Zhao, Yi Zhu, Xiaoshen Wang

**Affiliations:** Department of Radiation Oncology, Eye and ENT Hospital of Fudan University, Shanghai, China

**Keywords:** hypopharyngeal squamous cell carcinoma, TPF, chemotherapeutic sensitivity, glycolysis, immune response, SEC61G

## Abstract

**Background:** There is an urgent need to identify which patients would benefit from TPF chemotherapy in hypopharyngeal squamous cell carcinoma (HPSCC) and to explore new combinations to improve the treatment effect.

**Materials and methods:** Gene**-**expression profiles in 15 TPF-sensitive patients were compared to 13 resistant patients. Immunohistochemistry (IHC) was performed to detect CD8^+^ T cells in 28 samples. Patient-Derived Tumor Xenograft (PDX) model and IHC were used to verify markers that optimize treatment for HPSCC.

**Results:** Through RNA sequencing 188 genes were up-regulated in TPF chemotherapy-resistant (CR) tissues were involved in T cell activation, while 60 down-regulated genes were involved in glycolysis. Gene set enrichment analysis (GSEA) showed that chemotherapy-sensitive (CS) group upregulation of the pathways of glycolysis, while immune response was downregulated. CIBERSORT, MCP-counter, and IHC proved that most immune cells including CD8^+^ T cells in the CR significantly higher than that in CS group. Among the 16 up-regulated genes in CS had close associations, the most significant negative correlation between the gene level and CD8^+^ T cells existed in SEC61G. SEC61G was related to glycolysis, which was transcriptionally regulated by E2F1, and participated in antigen degradation through ubiquitin-dependent protein catabolic process. Palbociclib, combined with Cetuximab decreased the tumor burden and significantly suppressed the expression of E2F1 and SEC61G while activating MHC-I in PDX model.

**Conclusion:** Enhanced glycolysis promoted immune escape, but increased response to TPF chemotherapy. SEC61G was the center of the molecular network and targeting the E2F1/SEC61G pathway increased the expression level of MHC-I.

## Introduction

Head and neck squamous cell carcinoma (HNSCC) is one of the most common malignant tumors in the world. Hypopharyngeal squamous cell carcinoma (HPSCC) is relatively rare and accounts for roughly 3% of all head and neck cancers (Garneau et al., 2018; Zhang et al., 2021). Patients with HPSCC frequently present at an advanced stage which is characterized by extensive local spread and early metastasis (Uzcudun et al., 2001; Eckel and Bradley, 2019). HPSCC has a poor prognosis with an overall 5-year survival rate of only 25%–35% (Liu et al., 2020; Visini et al., 2021; Marzouki et al., 2022). Therefore, exploring effective targets to improve the prognosis of HPSCC is urgently required.

Surgery combined with radiotherapy and chemotherapy is the main treatment method, among which cisplatin (DDP)-based chemotherapy is widely applied in HPSCC (Yu et al., 2014; Li et al., 2022). However, chemotherapeutic resistance resulted in treatment failure, including recurrence and distant metastasis in various human tumors (Yu et al., 2014). Based on molecular mechanism, resistance to first-line chemotherapy agents, such as TPF as the most effective therapeutic management in HPSCC is a multifactorial event (Arora et al., 2010; Galluzzi et al., 2012; Mukhtar et al., 2014; Azwar et al., 2021).

Immunotherapy has dramatically changed the treatment landscape for patients with different tumors. Programmed death-ligand 1 (PD-L1) expression by tumor cells is a mechanism for down-regulation of antitumor T-cell responses and is a target for immunotherapy in various cancers. PD-1+ T lymphocytes were wildly infiltrated in HPSCC tissues, whose positivity in combination with CD8 high expression has been reported to present predictive potential in HPSCC (Hu et al., 2020; Wang et al., 2021). However, relevant large-scale and randomized clinical studies are lacking. There is an urgent need to identify which patients would benefit from chemotherapy or immunotherapy based on target markers, to optimize treatment for HPSCC patients in the future.

In previous study (Li et al., 2022), we have reported that modified TPF chemotherapy was an effective approach for laryngeal preservation in HPSCC. However, there were still 35%–45% of patients who showed no response after chemotherapy (Li et al., 2022). In present study, we further identify the differentially expressed genes that are closely related to the TPF-chemotherapy-sensitivity (CS) in HPSCC by RNA sequencing. Patient-Derived Tumor Xenograft (PDX) model and immunohistochemistry (IHC) staining were used to verify markers that optimize treatment for HPSCC.

## Materials and methods

### Patient source and inclusion criteria

Between April 2014 and December 2018, patients with a pathological diagnosis of HPSCC who were treated in the Eye and ENT Hospital of Fudan University were enrolled. The inclusion criteria were as follows: 1) Patients with a confirmed pathological diagnosis of primary HPSCC in our hospital, and the tumor specimens from the biopsy are available; 2) Patients were not exposed to any treatment before they got a biopsy; 3) Locally advanced HPSCC with confirmed clinical stages of III, IVA, and IVB as defined by the eighth edition of the American Joint Committee on Cancer; 4) Patients received at least 2 cycles of TPF induction chemotherapy, and the tumor regression could be evaluated; 5) Patients with complete clinical and follow-up data.

### TPF induction chemotherapy and treatment efficacy

We performed at least two 21-day cycles of TPF neoadjuvant chemotherapy with docetaxel (75 mg/m^2^, day 1), cisplatin (25 mg/m^2^, days 1–3), 5-fluorouracil (500 mg/m^2^, days 1–4) or capecitabine (825 mg/m^2^, twice daily, days 1–14). Treatment efficacy was evaluated based on Response Evaluation Criteria in Solid Tumors (RECIST, V1.1). The efficacy of induction chemotherapy was evaluated on days 14–21 of the second cycle of chemotherapy. According to the RECIST, a total of 28 patients met the screening criteria, and were divided into two groups: fifteen patients proved to be sensitive to treatment, while the rest thirteen patients were grouped into chemotherapy-resistant (CR). The clinical features are summarized in Supplementary Table S1.

### Gene quantitative profiling and bioinformatic analysis

The research was approved by the Clinical Ethics Committee and we obtained written informed consent from the patient. RNA sequencing was conducted by Shanghai oebiotech Co. (Shanghai, China). Very low expression genes were filtered out firstly (sum (FPKM) < 6, FPKM means Fragments Per Kilobase of transcript per Million fragments mapped), fold change (FC) combined with T-test were conducted to analyze the differentially expressed genes (DEGs). Compared to the gene expression from the sensitive group, FC < 1 in the resistant group was regarded as a down-regulated gene, while FC > 1 was up-regulated gene. Genes with a FC > 2, or <.5 compared to the sensitive group and *p* < .05 were subjected to further verification.

The gene ontology (GO), Kyoto encyclopedia of genes and genomes (KEGG) were analyzed using the online system “Metascape” (https://metascape.org) for enrichment analysis. R package clusterProfiler (3.8.0) (Yu et al., 2012) was used to carry out Gene Set Enrichment Analysis (GSEA) to elaborate on the significant pathway between drug-resistant and sensitive samples. C5.go.bp.v7.5.1.symbols.gmt, c2.cp.kegg.v7.5.1.symbols.gmt, and h.all.v7.5.1.symbols.gmt in the MsigDB Collections were used as the reference gene collection. FDR <.25, adjusted *p*-value <.05, and |NSE| > 1 was considered as statistically significant. Two methods were used to analyze the components of immune cells. Method 1: the relative proportions of 22 types of immune cells were analyzed by online tool CIBERSORT (https://cibersort.stanford.edu). Method 2: Based on the FPKM expression profile, the following cell abundance was analyzed using MCP-counter (https://zenodo.org/record/61372#.XVPIB6276qB) of R language: T cells, Monocytic lineage, B lineage, Neutrophils, Cytotoxic lymphocytes, NK cells, CD8^+^T cells, Myeloid dendritic cells, Fibroblasts, and Endothelial cells. The significance of different types of immune cells in the two groups was analyzed by Mann-Whitney *U* test. The online system “STRING (https://cn.string-db.org/)” was conducted to build the functional protein association network.

### Enrichment analysis of SEC61G

The correlation between the expression of different genes and CD8^+^T cells infiltration, or MHC-I molecular antigen presentation was explored by TIMER 2.0 (https://cistrome.shinyapps.io/timer/). The correlation between SEC61G expression and EGFR expression in HNSC normal and tumor was explored by GEPIA (http://gepia.cancer-pku.cn/). Datasets of Head and Neck Squamous Cell Carcinoma (TCGA, Firehose Legacy) was acquired from cBioportal (https://www.cbioportal.org/). Spearman’s correlation coefficient (r) was calculated to assess the correlation between co-expressed genes and SEC61G, of which *p* < .001 and |r| > .35 were selected. “Transcription Factor Target” analysis for 2275 co-expressed genes related to SEC61G in HNSCC (Head and Neck Squamous Cell Carcinoma (TCGA, Firehose Legacy)) was carried out through Webgestalt (http://www.webgestalt.org/). The gene expression data of SEC61G and the HPV status in TCGA-HNSC project were acquired from UCSC Xena browser (http://xenabrowser.net/datapages/). Cases without complete gene expression data and HPV status were excluded. Patients with HNSCC were classified into low- and high-expression groups according to the mean SEC61G expression value.

### Exploring the immune-related functional relationship network

ImmuNet (https://immunet.princeton.edu/) was utilized to predict the related molecular network in antigen processing and presentation, natural killer cell mediated cytotoxicity, B cell receptor signaling pathway, T cell receptor signaling pathway, and chemokine signaling pathway.

### *In vivo* study

Animal studies were performed in compliance with the International Animal Care and Use Committee-approved protocol (IACUC). PDX model, was derived from a patient with HPSCC who had never received systemic therapy. The study was approved by the Ethics Committee and the patient agreed with written informed consent. Small pieces (5 × 5 × 5 mm^3^) of tumor samples were obtained from the patient and subcutaneously injected into NOD-SCID mice. When tumor sizes reached 1,000 mm^3^, it was removed and divided into small pieces and transplanted into another mouse. We defined patient-oriented mice model as P0 generation, and subsequently generations were numbered consecutively (P1, P2, and P3). After P1 generation, the tumor was engrafted into BALB/c male nude mice. P3 generation model was utilized for drug response. The mice were randomly grouped and treatment started when tumor sizes reached 70 mm^3^. Each group included 6 mice.

Mice were treated with 100 mg/kg Palbociclib (daily), 1 mg Cetuximab (weekly), or a combination of Palbociclib and Cetuximab. Palbociclib was administrated by oral gavage and Cetuximab was administrated by intraperitoneal injection. Tumor size and body weight were measured twice weekly. After 28 days of drug treatment, tumors were removed, weighed, photographed, fixed, and kept at −80°C. (Palbociclib (PD-0332991) was obtained from Selleck Chemicals and dissolved in water. Cetuximab was obtained from Merck kGaA.)

### Immunohistochemistry

Antibodies against E2F1 (sc-251): Santa Cruz Biotechnology. SEC61G (11147-2-AP) and HLA class I ABC (15240-1-AP): Proteintech. CD8 (GTX16696): GeneTex. IHC staining was performed as described previously (Hu et al., 2020).

### Statistical analysis

Statistical analyses were conducted using GraphPad Prism (version 9.0, San Diego, CA, United States). Mann-Whitney U or ANOVA was used to compare the difference between groups. Fisher’s exact probability method was used for comparison between groups of categorical variables. All tests were on two sides, and *p* < .05 was considered statistically significant.

## Results

### Molecular profiles in TPF-responsive patients relative to resistant patients

The drug-sensitive one was defined as tumor volume reduced more than 50% after treatment. Among 15,671 protein-coding genes with a sum (FPKM) greater than 6, 1,310 genes exhibited statistically differentiated expression in the CR compared to CS (*p* < .05) (Supplementary Figure S1). Furthermore, of these 1,310 genes, 188 genes with an FC > 2 presented a higher expression in the tissue from the CR, while 60 with an FC < .5 were down-regulated (Supplementary Figure S1).

### Functional annotation of differentially expressed genes

**FIGURE 1:**
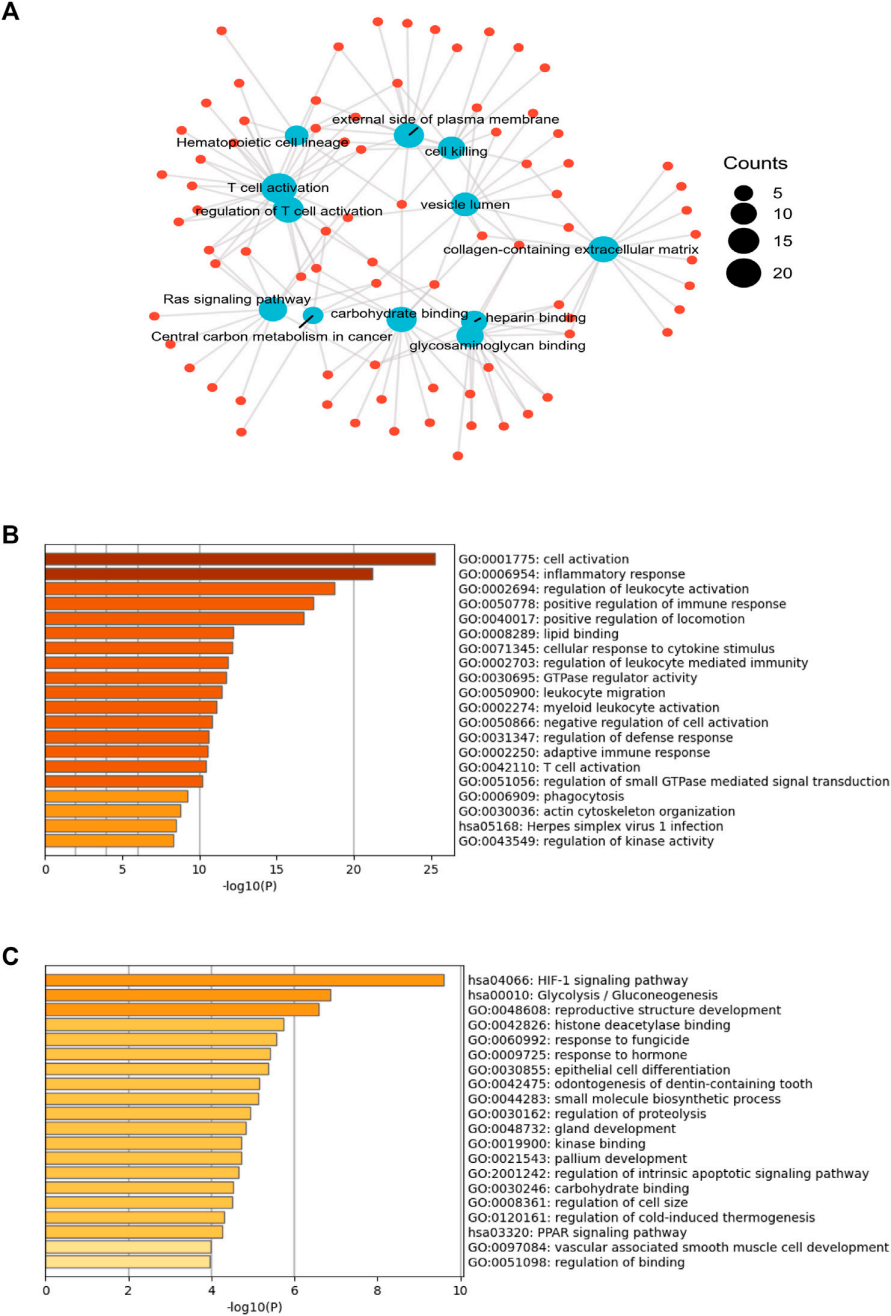
The gene expression profiles in chemotherapy-sensitive and resistant hypopharyngeal cancer patient tissues. **(A)** Gene oncology (GO)-biological processes (BP), GO-molecular functions (MF) and Kyoto encyclopedia of genes and genomes (KEGG) analysis of 1,310 genes showed significant differences in the expression levels of tissues from the non-sensitive group compared to the sensitive group (*p* < .05) based on mRNA-targeted genes. **(B)** The GO enrichment analysis and KEGG pathway analysis for evaluating 188 up-regulated genes in the resistant group. **(C)** The GO enrichment analysis and KEGG pathway analysis for evaluating 60 up-regulated genes in the sensitive group.

From the identified 1,310 differentially expressed genes, pathway enrichment analysis was performed. As shown in Figure 1A, “T cell activation”, and “Central carbon metabolism in cancer” attracted our attention. Next, the 188 up-regulated genes in resistant group (Figure 1B) were involved in “inflammatory response”, “regulation of leukocyte activation”, “positive regulation of immune response”, “adaptive immune response”, and “T cell activation”. On the contrary, 60 down-regulated genes were involved in “HIF-1 signaling pathway”, “Glycolysis/Gluconeogenesis”, and “regulation of proteolysis” (Figure 1C).

**FIGURE 2:**
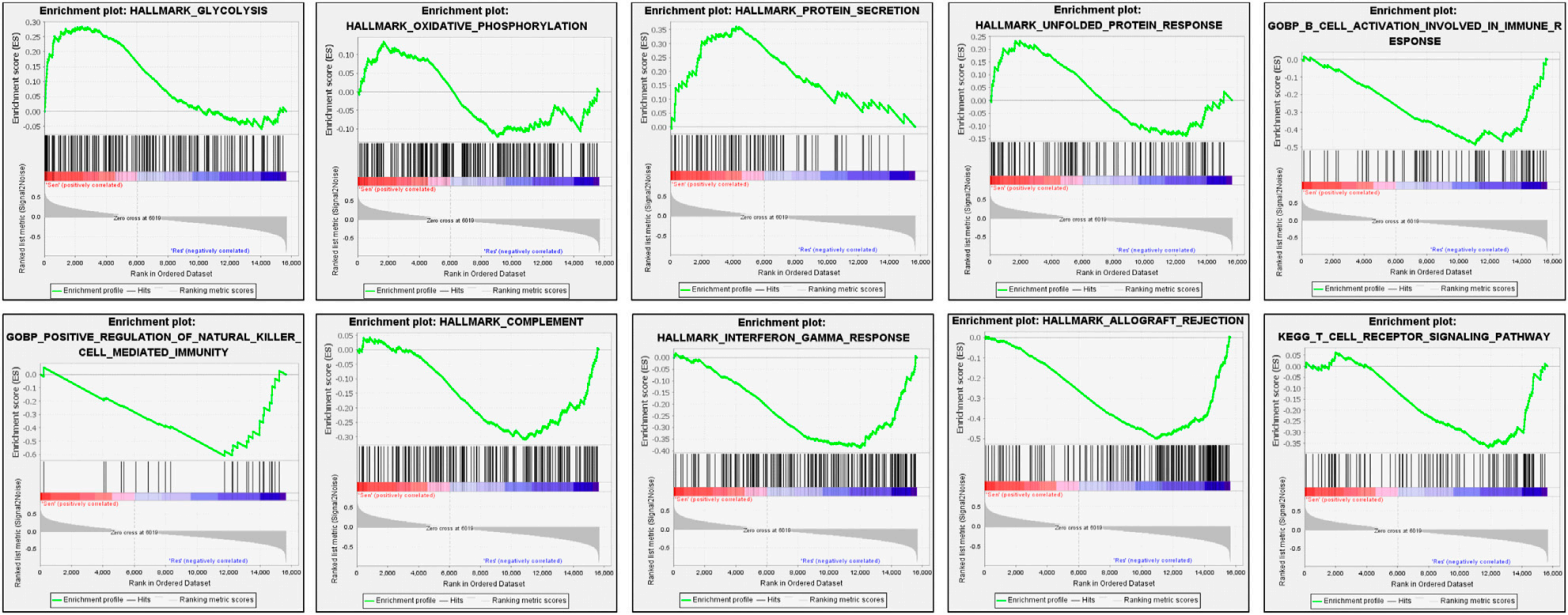
Enrichment plots derived from the gene set enrichment analysis (GSEA) related to drug-sensitive group.

A GSEA analysis (Figure 2) was also conducted to explore the potential pathways correlated with the CS group. GSEA analyses showed that CS group upregulation of the pathways involving glycolysis, oxidative phosphorylation, protein secretion, and unfolded protein response (UPR). However, B cell activation involved in immune response, positive regulation of natural killer cell mediated immunity, complement, interferon gamma response, allograft rejection, and T cell receptor signaling pathway were downregulated.

### The correlation between chemosensitivity and immune cell infiltration

**FIGURE 3:**
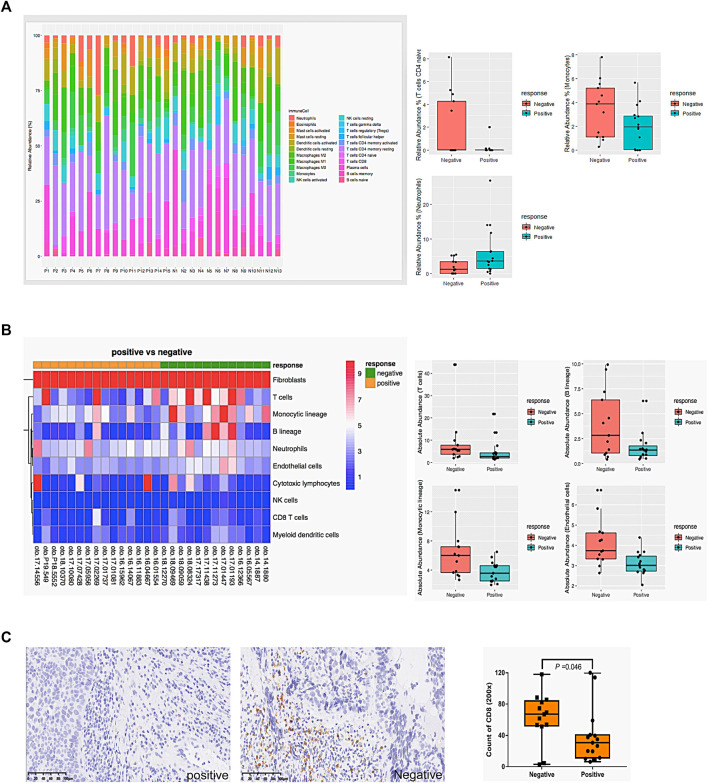
The correlation between chemosensitivity and immune cell infiltration. **(A)** The relative proportions of 22 types of immune cells in 28 samples were analyzed by online tool CIBERSORT. **(B)** The absolute abundance of 8 types of immune cells, endothelial cells, and fibroblasts in 28 samples was analyzed by MCP-counter. **(C)** Representative CD8 immunohistochemistry staining and the count of CD8 positive cells in hypopharyngeal cancer tissues. Mann-Whitney U was used to compare the difference between groups. Negative: drug-resistant; Positive: drug-sensitive.

Due to the pathways related to immune response being downregulated in CS group (Figures 1, 2), firstly, as shown in Figure 3A, CIBERSORT showed that the proportions of naïve CD4^+^T cells and monocytes significantly increased in the CR group (*p* < .05), while neutrophils significantly increased in the CS group (*p* < .05). Secondly, the absolute abundance of 8 types of immune cells, endothelial cells, and fibroblasts were analyzed by MCP-counter. As shown in Figure 3B, T cells, B cells, monocytes, and endothelial cells were significantly increased in the CR group (*p* < .05).

We further performed IHC to detect CD8^+^ T cells in 28 samples (Figure 3C). Consistent with our hypothesis, CD8^+^ T cells were significantly lower in resistant patients’ samples than in sensitive ones (*p* = .046). Supplementary Table S2 shows the correlation between CD8^+^lymphocyte subsets and clinicopathological parameters of 28 patients. The increased infiltration density of CD8^+^ T cells was significantly associated with CR (*p* = .002) but not other factors (*p* > .05).

### Key factor in HPSCC chemosensitivity

**FIGURE 4:**
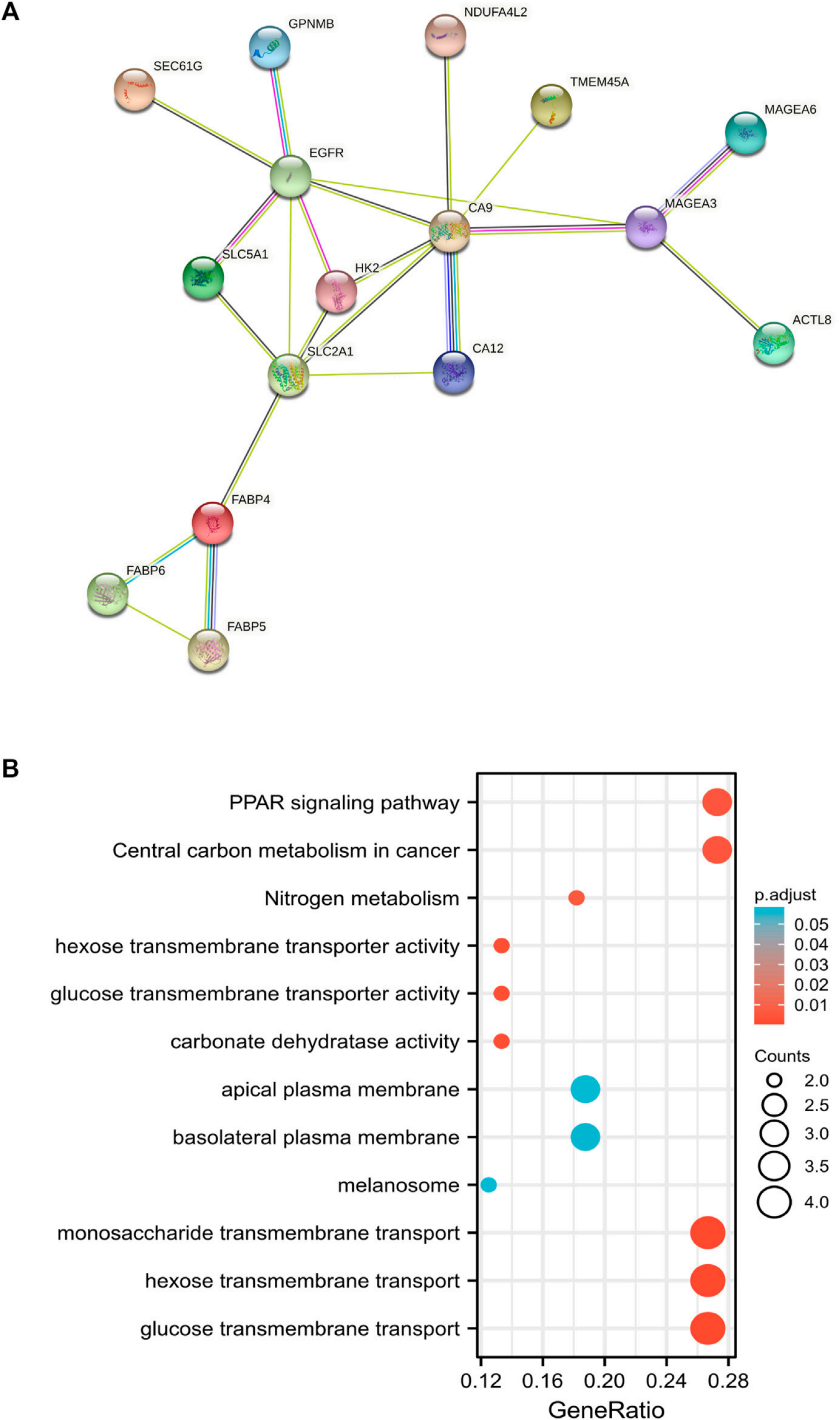
The protein association network related to up-regulated genes in chemotherapy-sensitive group. **(A)** Sixteen proteins among 60 up-regulated proteins in sensitive group which had functional and physical associations were predicted by STRING web platform. **(B)** GO and KEGG analyses of 16 related genes in functional protein association network.

STRING web platform was used to predict the protein association network related to 60 up-regulated genes in the CS group and we found that 16 proteins had functional and physical associations (Figure 4A). The 16 related proteins closely related to the regulation of glucose metabolic pathway and signaling (Figure 4B). Owing to the infiltration of CD8^+^ T cells being significantly decreased in CS group, among the 16 genes, CA12, EGFR, FABP4, FABP5, HK2, MAGEA3, MAGEA6, SEC61G, SLC2A1, SLC5A1, and TMEM45A were significantly negatively correlated with CD8^+^ T cells infiltration (Supplementary Figure S2), while the most significant negative correlation existed in SEC61G (partial correlation: −.273, *p* = 1.52e-09), also known as endoplasmic reticulum (ER) SEC61 gamma subunit.

### Functional enrichment analysis of SEC61G in HNSCC

**FIGURE 5:**
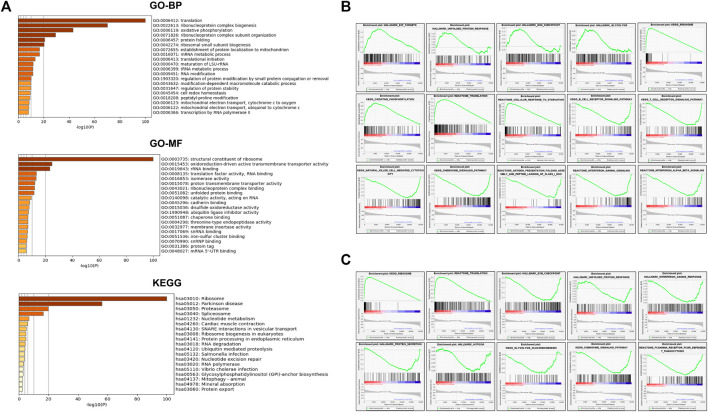
SEC61G-associated gene enrichment analysis. **(A)** Gene oncology (GO)-biological processes (BP), GO-molecular functions (MF) and Kyoto encyclopedia of genes and genomes (KEGG) analysis of 1041 SEC61G positive co-expression genes in HNSCC (Head and Neck Squamous Cell Carcinoma (TCGA, Firehose Legacy)). **(B)** Enrichment plots derived from the gene set enrichment analysis (GSEA) related to high SEC61G expression in HPV-negative HNSCC based on TCGA-HNSC database. **(C)** GSEA related to high SEC61G expression in HPV-positive HNSCC based on TCGA-HNSC database.

A total of 2275 co-expressed genes related to SEC61G in HNSCC (Head and Neck Squamous Cell Carcinoma (TCGA, Firehose Legacy)) were identified, of which 1,041 genes were positively correlated. Then the functions of positively co-expression in patients with HNSCC were predicted (Figure 5A). The GO-biological processes (BP), molecular functions (MF) items, and KEGG pathway were including “ribosome”, “oxidative phosphorylation”, “protein folding”, “UPR”, “proteasome”, “ubiquitin mediated proteolysis”.

Next, we divided HPV-negative and HPV-positive patients respectively into high- and low-expression groups based on the mean SEC61G expression. In HPV-negative HNSCC (Figure 5B), GSEA showed that high SEC61G expression positively upregulated the pathways including E2F targets, UPR, G2M checkpoint, glycolysis, ribosome, oxidative phosphorylation, and translation. However, B/T cell receptor signaling pathway, natural killer cell mediated cytotoxicity, antigen presentation folding assembly and peptide loading of class I MHC, and interferon gamma signaling were downregulated. Surprisingly, in HPV-positive HNSCC (Figure 5C), UPR, protein secretion, hypoxia, and glycolysis were downregulated.

### CDK4/6 inhibitor and EGFR inhibitor increase MHC-I expression by targeting E2F1/SEC61G axis

**FIGURE 6:**
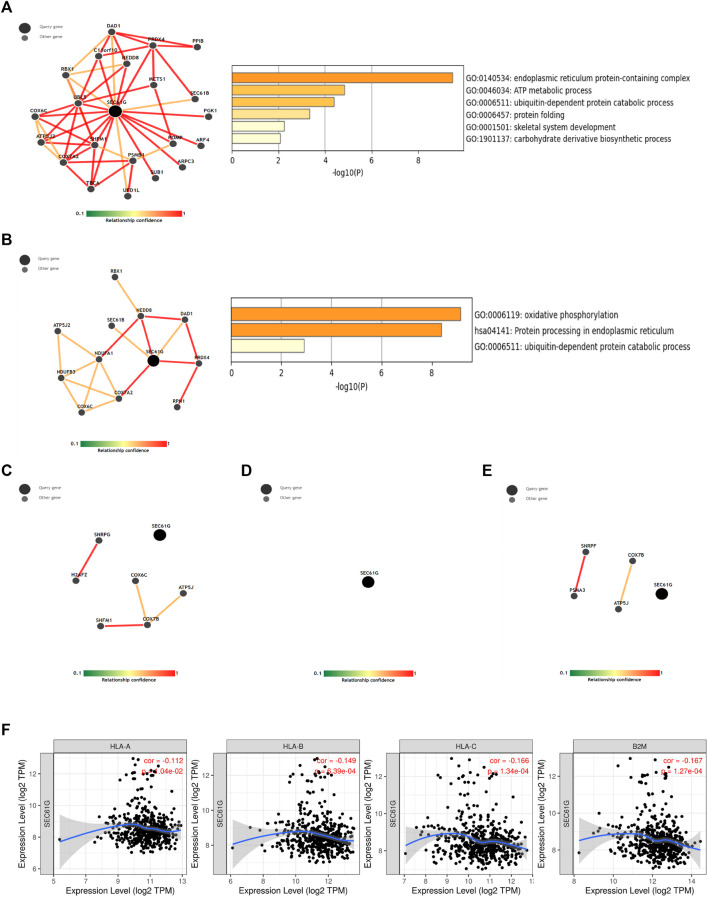
The immune-related analysis involving SEC61G. **(A)** A molecular network involving SEC61G in antigen processing and presentation based on ImmuNet analysis. **(B)** A molecular network involving SEC61G in natural killer cell mediated cytotoxicity based on ImmuNet analysis. **(C)** No molecular network was built related to B cell receptor signaling pathway. **(D)** No molecular network was built related to T cell receptor signaling pathway. **(E)** No molecular network was built related to chemokine signaling pathway. **(F)** The correlation between SEC61G expression and key components in MHC-I presentation in HNSCC *via* TIMER.

SEC61G acted a significant role in MHC-I mediated antigen processing and presentation (Figure 5). Thus, ImmuNet was utilized to explore the immune-related molecular network involving. As shown in Figures 6A,B, SEC61G was the center of the molecular network in relation to “antigen processing and presentation” and “natural killer cell mediated cytotoxicity”. Functional enrichment showed that endoplasmic reticulum protein-containing complex and ubiquitin-dependent protein catabolic process were enriched. However, neither molecular network nor enrichment was obtained regarding SEC61G in B/T cell receptor and chemokine signaling pathway (Figures 6C–E), which means that SEC61G is not directly involved in the inhibition of T cells signaling pathway. Figure 6F illustrated that SEC61G expression significantly negatively correlated with HLA-A, HLA-B, HLA-C, and β2-microglobulin (B2M) in HNSCC.

**FIGURE 7:**
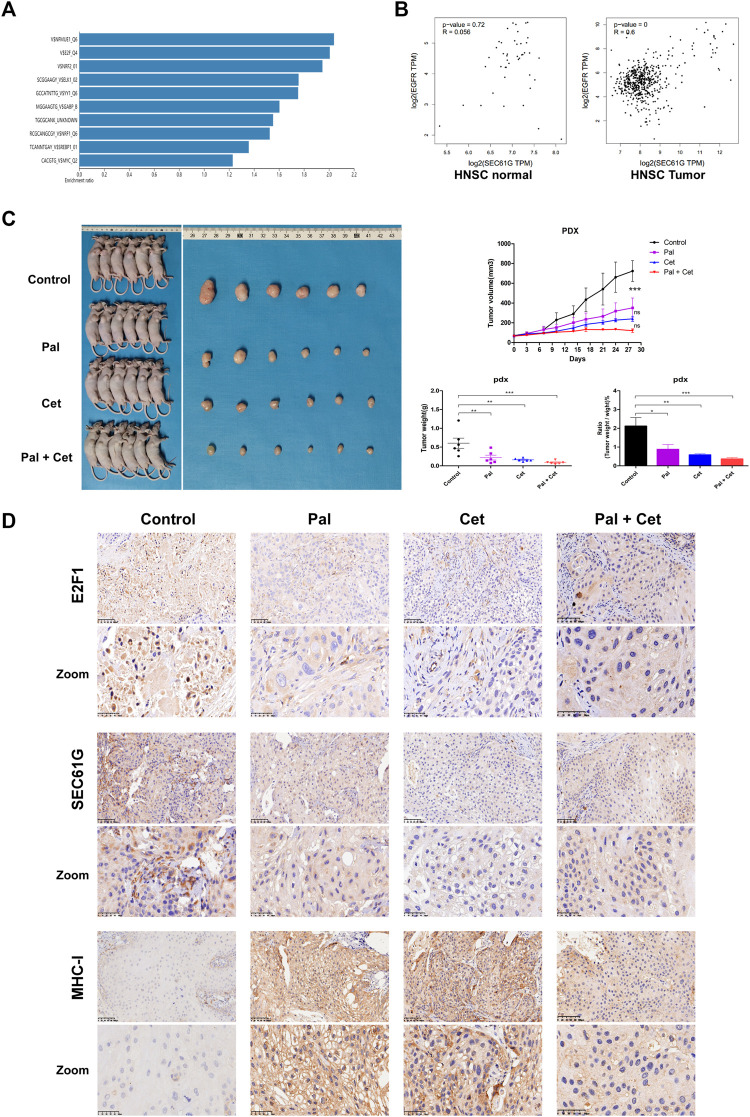
CDK4/6 inhibitor and EGFR inhibitor increase MHC-I expression by targeting E2F1/SEC61G axis. **(A)** “Transcription Factor Target” analysis for 2275 co-expressed genes related to SEC61G in HNSCC (Head and Neck Squamous Cell Carcinoma (TCGA, Firehose Legacy)) through webgestalt approach. **(B)** The correlation between the expression of SEC61G and EGFR in HNSC normal and HNSC tumor based on TCGA-HNSC database. **(C)** Combination Palbociclib and Cetuximab in Patient-Derived Tumor Xenograft (PDX) model related to hypopharyngeal squamous cell carcinoma. Hypopharyngeal cancer PDX models were treated with control, Palbociclib (100 mg/kg/day), Cetuximab (1 mg/week) or Cetuximab plus Palbociclib for 28 days (n = 6 per group). The growth curves of xenografts are shown. After 28 days, the mice were killed, and tumors were dissected, weighed and photographed. One-way ANOVA and Tukey’s multiple comparisons test. *p < .05, **p < .01, ***p < .001. Pal, Palbociclib; Cet, Cetuximab; ns, not significant. **(D)** Immunohistochemistry staining of E2F1, SEC61G, and HLA class I ABC (MHC-I) in PDX models after treatments. Pal, Palbociclib treatment mouse; Cet, Cetuximab treatment mouse; Pal + Cet, Palbociclib plus Cetuximab treatment mouse.

A “Transcription Factor Target” analysis was performed using the 2275 co-expressed genes related to SEC61G in HNSCC and predicted that SEC61G was transcriptionally regulated by E2F (Figure 7A). The result was consistent with the study in breast cancer, E2F1 bound to the promoter of SEC61G directly and controlled its expression (Ma et al., 2021). Previous reports have shown that SEC61G and EGFR were encompassed in the minimal overlapped regions of amplification (Lu et al., 2009), and inhibition of EGFR could decrease downstream E2F1 transcriptional activity (Nishioka et al., 2011; Wang et al., 2020). As illustrated in Figure 7B, SEC61G expression significantly positively correlated with EGFR in HNSC tumor but not in HNSC normal. Therefore, we believe that targeting E2F1/SEC61G by CDK4/6 inhibitor or EGFR inhibitor to improve the expression of MHC-I in HNSCC is feasible. To verify our hypothesis, we analyzed the potential application of Palbociclib/Cetuximab in PDX model of HPSCC (Figure 7C). Palbociclib monotherapy surprisingly exhibited a comparable effect to that in Cetuximab monotherapy. We observed a significant reduction in tumor growth in combination treatment. In the combination group, the mean tumor weight was lower (90 mg) compared to Palbociclib (230 mg), Cetuximab (170 mg) and vehicle control (600 mg). Next, the tumors in PDX model were analyzed by IHC. As expected, the levels of E2F1 and SEC61G were substantially diminished in Palbociclib-treated group when compared to control, while the expression of MHC-I was correspondingly increased (Figure 7D). Similarly, Cetuximab monotherapy or the combination therapy also effectively decreased the expression of E2F1 and SEC61G but activated MHC-I (Figure 7D).

## Discussion

The prognosis of HPSCC is extremely poor, especially metastasis is the main factor of poor prognosis which is widely known at present (Kuo et al., 2016). Chemotherapy plays a very important role, commonly used chemotherapy regimens for HPSCC include TPF. However, resistance to drugs is a common clinical issue in the treatment of patients with HPSCC. Many studies have confirmed single genetic markers associated with chemotherapy sensitivity in HPSCC. For instance, COX-2 expression has been found to be associated with chemoresistance through the cancer stemness property (Saito et al., 2021). The ctDNA might play a significant role in DDP resistance in HPSCC by amplifying related functional genes (Lin et al., 2022). However, genome-wide analysis associated with chemotherapy resistance HPSCC is lacking.

In this study, the main DEGs involving the BPs including “regulation of immune response” and “Glycolysis/Gluconeogenesis”. Glycolysis has been confirmed to be significantly associated with the development of certain diseases (Ganapathy-Kanniappan and Geschwind, 2013). The genetic modifications could influence metabolism and induced aerobic glycolysis (Van den Bossche et al., 2022). HNSCC presents a high rate of glycolysis to fulfill their metabolic requirements (Raj et al., 2021). Glycolysis provides ATP, NADPH, and carbon skeletons for the growth and construction of tumor cells (Dang, 2012; Alfarouk et al., 2014). As to the correlation between glycolysis and the response to the chemotherapy, most evidence illustrated that enhanced glycolysis contributed to resistance to cisplatin-based chemotherapy in many tumors (Simons et al., 2007; Xintaropoulou et al., 2018; Zhang et al., 2018; Sawayama et al., 2019; Dai et al., 2020; Varghese et al., 2020). However, in our study, we surprisingly found that DEGs significantly clustered in glycolysis which is associated with TPF-chemotherapy sensitivity in HPSCC patients. For example, SLC5A1, which was one of the 16 related up-regulated genes in CS group in our study, facilitates glucose transport (Mueckler and Thorens, 2013). A risk model found that SLC5A1 is one of the three hub genes that related to cisplatin therapy response in ovarian cancer (Chen et al., 2022). Several studies have also proved that an increased glycolysis rate can enhance the sensitivity to chemotherapy. As a key driver of aerobic glycolysis, upregulation of Pyruvate Kinase M2 (PKM2) facilitates the response to chemotherapy in gastric cancer, breast cancer and intestinal cancer (Zhu et al., 2016). Knockdown of PFKFB2 increased the glycolysis rate and enhanced the effect of paclitaxel-based chemotherapy in breast and ovarian cancers (Yang et al., 2019). Metformin suppressed Nrf2 and decreased cisplatin resistance through enhanced glucose metabolism (Cai et al., 2020). Hypoxia improved the response of retinoblastoma cells to chemotherapy by activation of glycolysis (Yang et al., 2017). Together, glycolysis is a complex process, which may display completely opposite effects in different settings.

Another case that surprised us was that most immune cells including CD8^+^ T cells in the CS group significantly lower than that in CR group. Glycolysis can transform the efficacy of immune cells and contributes to cancer cells to escape immunological surveillance within the tumor microenvironment (TME) (Gill et al., 2016). Disturbance of intracellular pH due to the lactate produced by glycolysis inhibits the proliferation and the activity of immune cells in the TME (Ganapathy-Kanniappan, 2017). Such low-pH TME has been proved to decrease the physiology of antigen-presenting cells (Romero-Garcia et al., 2016). Xie (Xie et al., 2021) proved that lactate produced by Notch1 signaling inhibits the activity of T cell and NK cell and leads to the immune escape of lung cancer. Aerobic glycolysis enhanced by EGFR signaling inhibits the efficacy of cytotoxic T cell in triple negative breast cancer cells (Lim et al., 2016). Thus, alteration of TME to reduce glycolysis and acidity may improve the effect of immunotherapy.

Immunotherapy, including anti-PD-1/PD-L1 and CTLA-4 has been more and more widely used in HNSCC (Pitt et al., 2016; Dogan et al., 2018; Fasano et al., 2022). And its combination with chemotherapy is currently under investigation to improve long-term survival prognosis for tumor patients due to the synergism mechanism such as activation of various innate immune pathways. Pembrolizumab plus chemotherapy has been the preferred choice for recurrent HNSCC, based on the bulky disease or CPS scores from patients (Burtness et al., 2019). In this study, the correlation between DEGs and infiltrating immune cells was verified, and interestingly in CR patients the DEGs significantly clustered in the immune-related GO terms involving T cells, B cells, and monocytes, while not in CS group. The IHC results also validated that CD8^+^ T cells in the CR group significantly increased. Previous studies showed that with certain types of cancer, patients have been resistant to chemotherapy could be rescued by immunotherapy (Naoum et al., 2018; Yang et al., 2020), and our study probably provided the potential mechanism to identify the better immunotherapy combinations for patients based on different chemo-respond and different proportions of immunological cells infiltration.

The SEC61 complex forms a transmembrane channel where proteins are translocated across and integrated into the ER membrane (Hartmann et al., 1994; Greenfield and High, 1999). For kidney cancer SEC61G knockdown significantly promoted cell apoptosis in a caspase-dependent manner (Meng et al., 2021). Upregulation of SEC61G also promote cell invasion, and migration *via* modulating glycolysis in breast cancer (Ma et al., 2021). SEC61G expression is also elevated in head and neck cancer based on TCGA database, which is found to be significantly correlated with clinical stage, genetic mutation status, and poorer prognosis (Liang et al., 2021). However, its role in chemotherapy of HPSCC is unclear. In this study, SEC61G was up-regulated remarkably in CS group, meanwhile, the expression level of SEC61G was significantly negatively correlated with the infiltration of CD8^+^ T cells in our study. SEC61G is significantly involved in antigen processing. However, neither molecular network nor enrichment was obtained regarding SEC61G in B/T cell receptor signaling. It means that SEC61G is not directly involved in the inhibition of T cells infiltration. Without MHC-I molecules, ineffectively immune cell recruitment and activation would lead to tumor immune escape (Garrido et al., 2016). The study furtherly examined that Palbociclib, as the selective CDK4/6 inhibitor, combined with Cetuximab decreased the tumor burden in PDX model. E2F1, as the transcription factor directly bound to the promoter and regulate the expression of SEC61G, has been validated in breast cancer (Ma et al., 2021). Here we demonstrated that the expression of E2F1 and SEC61G were remarkably reduced, while the expression of MHC-I was increased. The combination therapy of immunotherapy and CDK4/6 inhibitors or EGFR inhibitors is rational. The role and mechanism of SEC61G modulating the chemosensitivity of HPSCC through metabolic and immune-related signaling pathways deserves further study.

Although this study enhanced a better understanding of the relationship between immune escape and the response to TPF chemotherapy, some limitations really existed. The exploration of the role of SEC61G was mainly based on RNA sequencing analysis and bioinformatic analysis, which lacks verification from samples, and the relevant pathways are still needed for further validation.

## Conclusion

In conclusion, enhanced glycolysis promoted immune escape, but increased response to TPF chemotherapy. SEC61G was the center of the molecular network and targeting the E2F1/SEC61G pathway increased the expression level of MHC-I, which potentially in turn affects the difference in tumor drug sensitivity. The molecular mechanisms that affect drug sensitivity in HPSCC deserve further exploration.

## Data Availability

The data presented in the study are deposited in the Genome Sequence Archive (GSA) repository (http://bigd.big.ac.cn/gsa-human), accession number HRA005361.

## Abbreviations

BP, biological processes; B2M, β2-microglobulin; DEGs, differently expressed genes; CR, chemo-resistant; CS, chemo-sensitive; CT, computed tomography; HNSCC, head and neck squamous cell carcinoma; ER, endoplasmic reticulum membrane; HPSCC, hypopharyngeal squamous cell carcinoma; DDP, cisplatin; PD-L1, programmed death-ligand 1; PDX, patient-Derived Tumor Xenograft; MRI, Magnetic resonance imaging; GO, gene ontology; KEGG, Kyoto encyclopedia of genes and genomes; GSEA, Gene Set Enrichment Analysis; IHC, immunohistochemistry; GLUT1, glucose transporter 1; PKM2, Pyruvate Kinase M2; TME, tumor microenvironment.
