# Supplementary material for: Increased response to TPF chemotherapy promotes immune escape in hypopharyngeal squamous cell carcinoma
Source: Front Pharmacol. 2023 Jan 13;13:1097197. doi: 10.3389/fphar.2022.1097197 (PMC9880322; doi:10.3389/fphar.2022.1097197)
Supplement: Supplementary file 1 [file DataSheet4.docx]

**Figure 1**

**Figure 1A** **The relevant code for R package**

library(clusterProfiler)

library(org.Hs.eg.db) ## org.Mm.eg.db

gene_ids = bitr(geneID = gene_list, fromType = "SYMBOL",

toType = "ENTREZID", OrgDb = "org.Hs.eg.db")

head(gene_ids)

# SYMBOL ENTREZID

# 1 NAT1 9

# 2 ADH1B 125

# 3 BIRC5 332

# 4 AQP9 366

# 5 BCL2A1 597

# 6 BMP4 652

ego <- enrichGO(gene = gene_ids$ENTREZID, OrgDb = "org.Hs.eg.db",

keyType = "ENTREZID", ont = "BP")

# #

# # over-representation test

# #

# #...@organism Homo sapiens

# #...@ontology BP

# #...@keytype ENTREZID

# #...@gene chr [1:209] "9" "125" "332" "366" "597" "652" "730" "771" "776" "820" "890" "891" "983" "991" "1062" "1101" "1111" "1307" "1308" "1311" ...

# #...pvalues adjusted by 'BH' with cutoff <0.05

# #...187 enriched terms found

# 'data.frame': 187 obs. of 9 variables:

# $ ID : chr "GO:0140014" "GO:0000280" "GO:0048285" "GO:0000070" ...

# $ Description: chr "mitotic nuclear division" "nuclear division" "organelle fission" "mitotic sister chromatid segregation" ...

# $ GeneRatio : chr "32/196" "34/196" "35/196" "23/196" ...

# $ BgRatio : chr "264/18670" "407/18670" "449/18670" "151/18670" ...

# $ pvalue : num 7.53e-25 4.63e-21 1.10e-20 2.23e-20 2.61e-19 ...

# $ p.adjust : num 2.33e-21 7.16e-18 1.14e-17 1.72e-17 1.61e-16 ...

# $ qvalue : num 2.01e-21 6.16e-18 9.78e-18 1.48e-17 1.39e-16 ...

# $ geneID : chr "332/652/891/991/1062/1111/3832/3833/4085/4605/4751/6790/7272/9055/9212/9232/9319/9493/9787/10403/10460/11065/22"| __truncated__ "332/652/891/991/1062/1111/3832/3833/4085/4605/4751/6790/7153/7272/9055/9212/9232/9319/9493/9787/10403/10460/110"| __truncated__ "332/652/891/991/1062/1111/3832/3833/4085/4137/4605/4751/6790/7153/7272/9055/9212/9232/9319/9493/9787/10403/1046"| __truncated__ "891/991/1062/3833/4085/4751/7272/9055/9212/9232/9319/9493/9787/10403/10460/23397/24137/51203/55143/64151/81620/81930/146909" ...

# $ Count : int 32 34 35 23 24 28 20 24 17 19 ...

# #...Citation

# Guangchuang Yu, Li-Gen Wang, Yanyan Han and Qing-Yu He.

# clusterProfiler: an R package for comparing biological themes among

# gene clusters. OMICS: A Journal of Integrative Biology

# 2012, 16(5):284-287

#### enrichKEGG(gene = gene_ids$ENTREZID, organism = "hsa", keyType = "ENTREZID")

### dot plot

dotplot(ego)

### cnet plot

cnetplot(ego)

**Figure 1B** **and 1C**

**The figures were created by online system “Metascape” (**[**https://metascape.org**](https://metascape.org)**).**

**Figure 2**

**The figure was created by the software of Gene Set Enrichment Analysis (GSEA) (4.1.0).**

**Figure 3**

**Figure 3A**

**The figure was created by online tool CIBERSORT (**[**https://cibersort.stanford.edu**](https://cibersort.stanford.edu)**).**

**Figure 3B The relevant code for R package**

library(MCPcounter)

input <- read.table("data/sample_input.txt",

header=T, row.names = 1, check.names=F)

probesets <- read.table("data/MCPcounter_probesets.txt",

sep="\t",stringsAsFactors=FALSE,

colClasses="character")

genes <- read.table("data/MCPcounter_genes.txt",

sep="\t",stringsAsFactors=FALSE,

header=TRUE,colClasses="character",

check.names=FALSE)

MCPcounterScore <- MCPcounter.estimate(input, featuresType = "HUGO_symbols", probesets = probesets,genes = genes)

MCPcounterScore <- normalize(MCPcounterScore)

write.table(MCPcounterScore, file = "data/MCPcounterScore.txt",sep="\t", quote=F, col.names=T)

annotation_col <- data.frame(colnames(MCPcounterScore))

colnames(annotation_col) <- "sample"

rownames(annotation_col) <- colnames(MCPcounterScore)

pheatmap(MCPcounterScore,show_colnames = F,cluster_rows = F,

cluster_cols = T,annotation_col = annotation_col,

cellwidth=15,cellheight=15,fontsize=5,

filename = 'figures/MCPcounter-heatmap.tiff')

**Figure 4**

**Figure 4A**

**The figure was created by online system “STRING (**[**https://cn.string-db.org/)**](https://cn.string-db.org/))**”.**

**Figure 4B The relevant code for R package**

library(clusterProfiler)

library(org.Hs.eg.db) ## org.Mm.eg.db

gene_ids = bitr(geneID = gene_list, fromType = "SYMBOL",

toType = "ENTREZID", OrgDb = "org.Hs.eg.db")

head(gene_ids)

# SYMBOL ENTREZID

# 1 NAT1 9

# 2 ADH1B 125

# 3 BIRC5 332

# 4 AQP9 366

# 5 BCL2A1 597

# 6 BMP4 652

ego <- enrichGO(gene = gene_ids$ENTREZID, OrgDb = "org.Hs.eg.db",

keyType = "ENTREZID", ont = "BP")

# #

# # over-representation test

# #

# #...@organism Homo sapiens

# #...@ontology BP

# #...@keytype ENTREZID

# #...@gene chr [1:209] "9" "125" "332" "366" "597" "652" "730" "771" "776" "820" "890" "891" "983" "991" "1062" "1101" "1111" "1307" "1308" "1311" ...

# #...pvalues adjusted by 'BH' with cutoff <0.05

# #...187 enriched terms found

# 'data.frame': 187 obs. of 9 variables:

# $ ID : chr "GO:0140014" "GO:0000280" "GO:0048285" "GO:0000070" ...

# $ Description: chr "mitotic nuclear division" "nuclear division" "organelle fission" "mitotic sister chromatid segregation" ...

# $ GeneRatio : chr "32/196" "34/196" "35/196" "23/196" ...

# $ BgRatio : chr "264/18670" "407/18670" "449/18670" "151/18670" ...

# $ pvalue : num 7.53e-25 4.63e-21 1.10e-20 2.23e-20 2.61e-19 ...

# $ p.adjust : num 2.33e-21 7.16e-18 1.14e-17 1.72e-17 1.61e-16 ...

# $ qvalue : num 2.01e-21 6.16e-18 9.78e-18 1.48e-17 1.39e-16 ...

# $ geneID : chr "332/652/891/991/1062/1111/3832/3833/4085/4605/4751/6790/7272/9055/9212/9232/9319/9493/9787/10403/10460/11065/22"| __truncated__ "332/652/891/991/1062/1111/3832/3833/4085/4605/4751/6790/7153/7272/9055/9212/9232/9319/9493/9787/10403/10460/110"| __truncated__ "332/652/891/991/1062/1111/3832/3833/4085/4137/4605/4751/6790/7153/7272/9055/9212/9232/9319/9493/9787/10403/1046"| __truncated__ "891/991/1062/3833/4085/4751/7272/9055/9212/9232/9319/9493/9787/10403/10460/23397/24137/51203/55143/64151/81620/81930/146909" ...

# $ Count : int 32 34 35 23 24 28 20 24 17 19 ...

# #...Citation

# Guangchuang Yu, Li-Gen Wang, Yanyan Han and Qing-Yu He.

# clusterProfiler: an R package for comparing biological themes among

# gene clusters. OMICS: A Journal of Integrative Biology

# 2012, 16(5):284-287

#### enrichKEGG(gene = gene_ids$ENTREZID, organism = "hsa", keyType = "ENTREZID")

### dot plot

dotplot(ego)

### cnet plot

cnetplot(ego)

**Figure 5**

**Figure 5A**

**The figure was created by online system “Metascape” (**[**https://metascape.org**](https://metascape.org)**).**

**Figure 5B and 5C**

**The figures were created by the software of Gene Set Enrichment Analysis (GSEA) (4.1.0).**

**Figure 6**

**Figure 6A, 6B, 6C, 6D, and 6E**

**The figures were created by online system “ImmuNet” (**[**https://immunet.princeton.edu/**](https://immunet.princeton.edu/)**).**

**Figure 6F**

**The figure was created by online system “TIMER 2.0” (https://cistrome.shinyapps.io/timer/).**

**Figure 7**

**Figure 7A**

**The figure was created by online system “Webgestalt” (**[**http://www.webgestalt.org/**](http://www.webgestalt.org/)**).**

**Figure 7B**

**The figure was created by online system “GEPIA” (http://gepia.cancer-pku.cn/).**
