# Supplementary material for: Increased response to TPF chemotherapy promotes immune escape in hypopharyngeal squamous cell carcinoma
Source: Front Pharmacol. 2023 Jan 13;13:1097197. doi: 10.3389/fphar.2022.1097197 (PMC9880322; doi:10.3389/fphar.2022.1097197)
Supplement: Supplementary file 2 [file Table1.docx]

Supplementary Table 1. Clinical data of 28 patients for microarray.

| **Group** | **No.** | **Sex** | **Age** | **Subsite** | **Stage** | **T stage** | **N stage** | **Differentiation degree** |
| --- | --- | --- | --- | --- | --- | --- | --- | --- |
| 0 | 1 | M | 65 | PS | IVB | 4a | 3 | moderate |
| 0 | 2 | M | 72 | PPW | III | 3 | 0 | moderate |
| 0 | 3 | M | 43 | PS | IVA | 3 | 2 | moderate |
| 0 | 4 | M | 62 | PPW | III | 3 | 1 | poor |
| 0 | 5 | M | 62 | PS | IVB | 4a | 3 | unknown |
| 0 | 6 | M | 61 | PPW | III | 2 | 1 | unknown |
| 0 | 7 | M | 65 | PPW | IVA | 3 | 2 | moderate |
| 0 | 8 | M | 63 | PS | IVA | 4a | 1 | moderate |
| 0 | 9 | M | 65 | PS | IVA | 2 | 2 | unknown |
| 0 | 10 | M | 71 | PS | IVA | 2 | 2 | unknown |
| 0 | 11 | M | 68 | PC | IVA | 2 | 2 | high |
| 0 | 12 | M | 57 | PPW | IVB | 4b | 2 | moderate |
| 0 | 13 | M | 61 | PS | IVA | 3 | 2 | unknown |
| 0 | 14 | M | 65 | PPW | III | 2 | 1 | moderate |
| 0 | 15 | M | 53 | PS | IVA | 4a | 2 | unknown |
| 1 | 16 | M | 55 | PPW | IVA | 4a | 2 | moderate |
| 1 | 17 | M | 65 | PS | IVA | 4a | 2 | moderate |
| 1 | 18 | M | 58 | PC | IVA | 3 | 2 | moderate |
| 1 | 19 | M | 60 | PS | III | 2 | 1 | moderate |
| 1 | 20 | M | 55 | PPW | IVA | 4a | 2 | unknown |
| 1 | 21 | M | 59 | PS | IVB | 3 | 3 | moderate |
| 1 | 22 | M | 51 | PS | IVA | 3 | 2 | moderate |
| 1 | 23 | M | 59 | PS | IVA | 2 | 2 | high |
| 1 | 24 | M | 65 | PS | IVA | 2 | 2 | moderate |
| 1 | 25 | M | 67 | PPW | IVB | 3 | 3 | moderate |
| 1 | 26 | M | 61 | PPW | IVA | 3 | 2 | high |
| 1 | 27 | M | 58 | PPW | IVB | 3 | 3 | moderate |
| 1 | 28 | M | 58 | PS | IVA | 3 | 2 | moderate |

Group 0, chemotherapy-sensitive group; Group 1, chemotherapy-resistant group; PS, pyriform sinus; PPW, posterior pharyngeal wall; PC, postcricoid region.
