# Supplementary figures and images for: Increased response to TPF chemotherapy promotes immune escape in hypopharyngeal squamous cell carcinoma
Source: Front Pharmacol. 2023 Jan 13;13:1097197. doi: 10.3389/fphar.2022.1097197 (PMC9880322; doi:10.3389/fphar.2022.1097197)

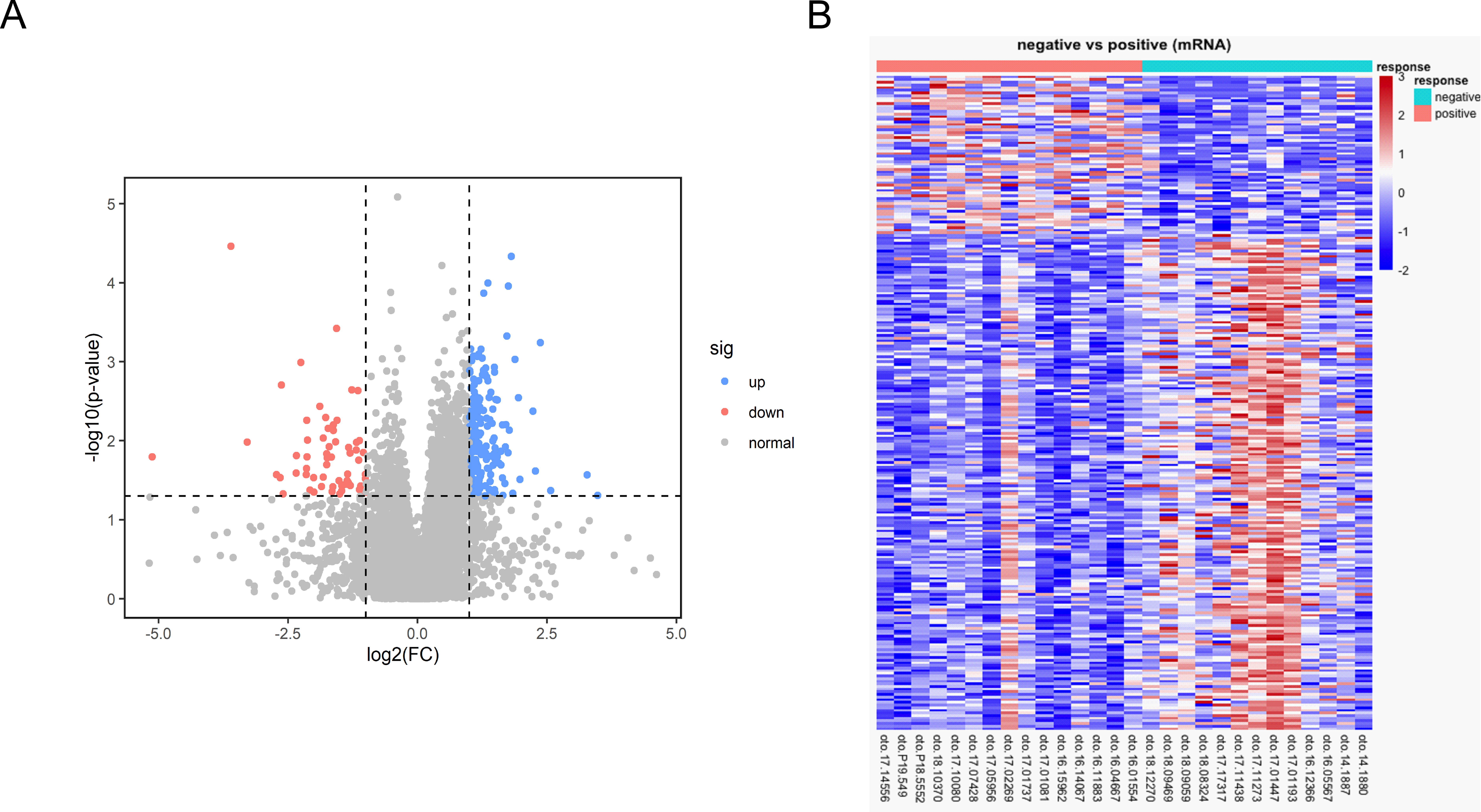

Supplement: Supplementary file 3 [file Image1.JPEG]

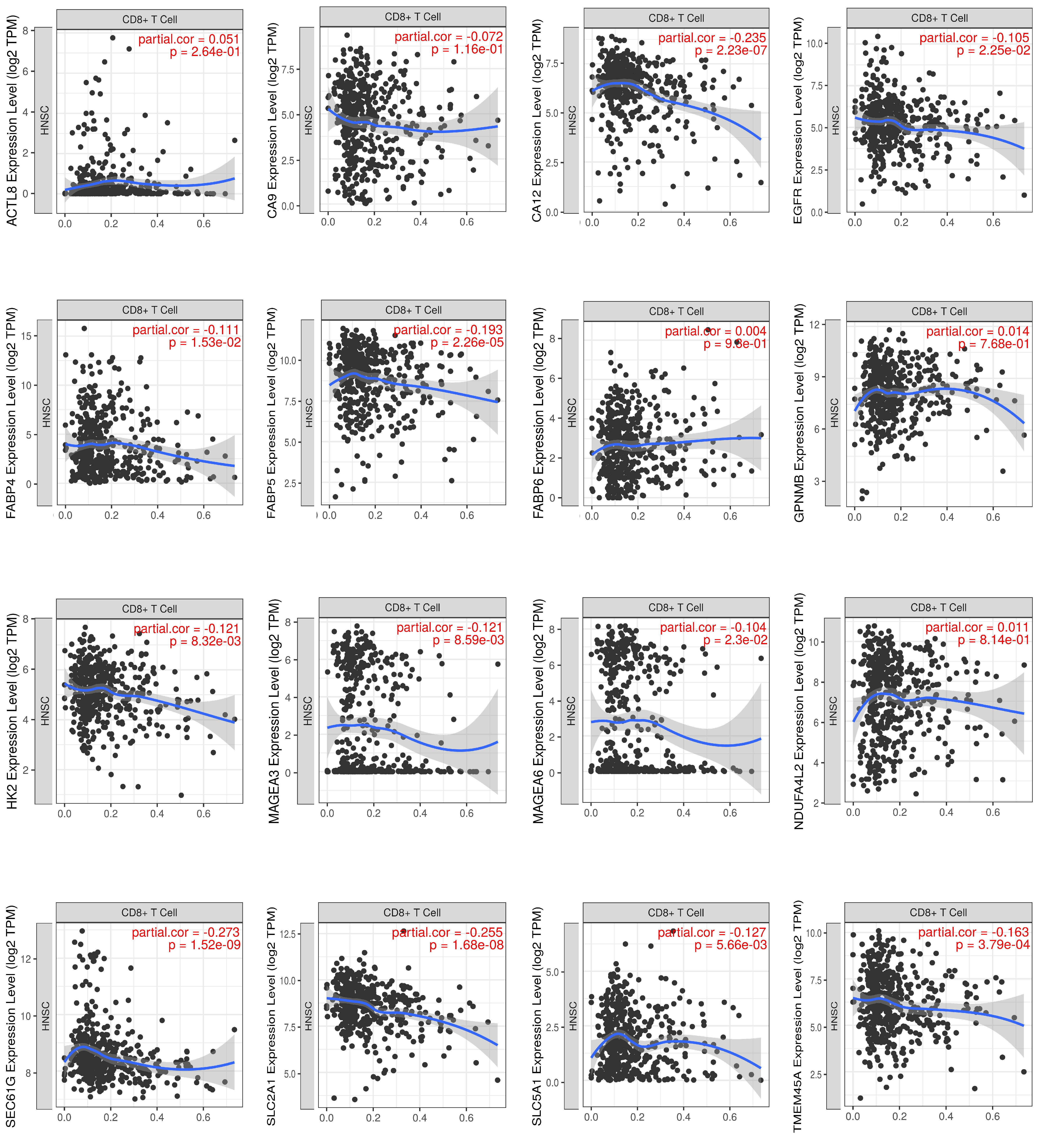

Supplement: Supplementary file 4 [file Image2.JPEG]
