# Supplementary material for: Increased response to TPF chemotherapy promotes immune escape in hypopharyngeal squamous cell carcinoma
Source: Front Pharmacol. 2023 Jan 13;13:1097197. doi: 10.3389/fphar.2022.1097197 (PMC9880322; doi:10.3389/fphar.2022.1097197)
Supplement: Supplementary file 5 [file DataSheet5.docx]

**Supplementary Figure captions**

**Supplementary Figure 1** The volcano plot **(A)** and hierarchical clustering **(B)** of differentially expressed mRNAs in drug-resistant (negative) group and drug-sensitive (positive) group.

**Supplementary Figure 2** The correlation between 16 related genes in sensitive group and CD8^+^ T cells was explored by TIMER 2.0 based on TCGA-HNSC database.
