# Supplementary material for: Increased response to TPF chemotherapy promotes immune escape in hypopharyngeal squamous cell carcinoma
Source: Front Pharmacol. 2023 Jan 13;13:1097197. doi: 10.3389/fphar.2022.1097197 (PMC9880322; doi:10.3389/fphar.2022.1097197)
Supplement: Supplementary file 7 [file Table2.docx]

Supplementary Table 2. The correlation between CD8^+^lymphocyte subsets

and clinicopathological parameters.

| **Characteristic** |  | **CD8** | |  | ***p*** |
| --- | --- | --- | --- | --- | --- |
|  |  | **low** | **High** |  |  |
| Age |  |  |  |  | 0.054 |
| ≤ 60 |  | 3 | 9 |  |  |
| > 60 |  | 11 | 5 |  |  |
| Smoking history |  |  |  |  | 1 |
| No |  | 1 | 2 |  |  |
| Yes |  | 13 | 12 |  |  |
| Alcohol history |  |  |  |  | 1 |
| No |  | 3 | 3 |  |  |
| Yes |  | 11 | 11 |  |  |
| Subsite |  |  |  |  | 1 |
| PS |  | 8 | 7 |  |  |
| Non-PS |  | 8 | 7 |  |  |
| Stage |  |  |  |  | 0.648 |
| III-IVA |  | 12 | 10 |  |  |
| IVB |  | 2 | 4 |  |  |
| T stage |  |  |  |  | 1 |
| T2-3 |  | 10 | 10 |  |  |
| T4a-4b |  | 4 | 4 |  |  |
| N stage |  |  |  |  | 0.648 |
| N0-1 |  | 4 | 2 |  |  |
| N2-3 |  | 10 | 12 |  |  |
| Chemotherapy  response |  |  |  |  | **0.002** |
| Positive |  | 12 | 3 |  |  |
| Negative |  | 2 | 11 |  |  |

Positive, chemotherapy-sensitive patients;

Negative, chemotherapy-resistant patients; PS, pyriform sinus.
